# An interdisciplinary approach to study individuality in biological and physical systems functioning

**DOI:** 10.1038/srep29512

**Published:** 2016-07-14

**Authors:** V. P. Mygal, A. V. But, G. V. Mygal, I. A. Klimenko

**Affiliations:** 1 Department of Physics, National Aerospace University, 61070 Kharkiv, Ukraine

## Abstract

Signals of system functioning of different nature are presented in the parameter space (state-velocity-acceleration) as a trajectory of dynamic events. Such signals geometrization allows to reveal the hidden spatio-temporal correlation in dynamics of systems functioning. It is shown that the nature of relationship between the dynamic parameters of signal determines the natural cycle of sensor functioning. Its restructuring displays the inherited features of systems functioning in signature package. The universal differential-geometry parameters and new integrative indexes of system functioning are used to analyze the signatures of biological and physical signals.

There are many common features in dynamical behavior of complex systems, both organic and inorganic^1^. Individuality of these systems functioning generally arises under extreme conditions. Safety and reliability of technical systems functioning depends on the set of interrelated factors. Among them the key factors are consistency and compatibility of all subsystems functioning. For instance, inconsistency between external factors and dynamics of internal processes leads to ambiguity and artifacts in response from semiconductor sensors^2^. From the other hand, the consistency of internal and external factors during semiconductor materials production can form unique photoelectric characteristics and phenomena^3^,^4^,^5^,^6^.

Behavior of biological systems is genetically inherited. System analysis is often ambiguous because of information about system functioning is hidden in combination of different signals^7^. For instance, the variety of shapes, spatial-temporal instability and cyclical rearrangement of structure are inherent to the physiological signals (ECG, etc.)^7^. Therefore ergonomists, biophysics, engineers, physicians, physicists, etc. have to use different approaches, models, signal processing techniques, etc. to analyze the reliability of various systems and their subsystems. The search for universal tools currently is very important for safety of manned systems. The main problems in express-identification and monitoring of psychophysiological state of a man (pilots, operators, dispatchers and decision-makers) are not solved. Thus, the application of modern signal processing tools used to analyze physiological signals are not allowed to avoid the problems (ambiguity, inconsistency, and others) associated with functional diagnostics of pilots, sportsmen and others. Therefore, to ensure the safety of manned systems functionality the search of universal tools for functioning signal analysis is very important.

Signals from various sensors in biological and technical systems usually have non-linear and cyclic character. Therefore, representation of physiological signals and sensor responses in biological and technical systems in a form of graphic images (phase portrait^8^, wavelet spectrogram^9^, complex plane^10^, graphical signature^11^, and others) turned out to be diagnostically informative. In this case, individuality in signals of subsystems functioning is most evident as a result of geometrization in a form of phase space signatures^12^. Signature configuration consists of closed sequence of geometrically ordered sections. It makes various signatures similar to cycles by presentation form. However, the configurations of signature-cycles even virtually identical signals are individual. Thus, among the variety of signature of dynamic I-V curves of semiconductor sensors and biosensors the similar configurations are meet^13^.

J. Maxwell and W. Thomson proposed the universal system of units (CGS) that based on length (L), time (T) and mass (M) units. In^14^, with a reference to Kepler’s third law, it was suggested that a unit of mass can be measured in m^3^/s^2^. But LT system of physical quantities was created by R. di Bartini^15^. He also suggested a neat way of physical quantities geometrization by means of their presentation in a form of [L^*l*^T^*k*^], where *l* + *k* ≤ 3 (*l, к* are integers). In the suggested table of physical quantities all of them are connected to each other through the differentiation and integration operations. This led us to the idea that in this way it is possible to represent the results of signals geometrization from different by nature biological and physical systems. Indeed, geometric similarities of dynamic components of signals are found in various sensors^12^,^13^,^16^. Individuality in sensor functioning is evident in signal’s signature configuration, i.e. after signal geometrization. The area spanned by signature is also informative. This area can be represented as a power of subset of possible system microstates^16^. Consequently, the signatures of signals from sensors and biosensors can be analyzed by complementary statistical and dynamical methods. This study is focuses on further development of interdisciplinary approach and search for universal tools. These are necessary for system analysis and control the complex systems functioning.

## Sensor response transformation into a subset of microstates

**Figure 1:**
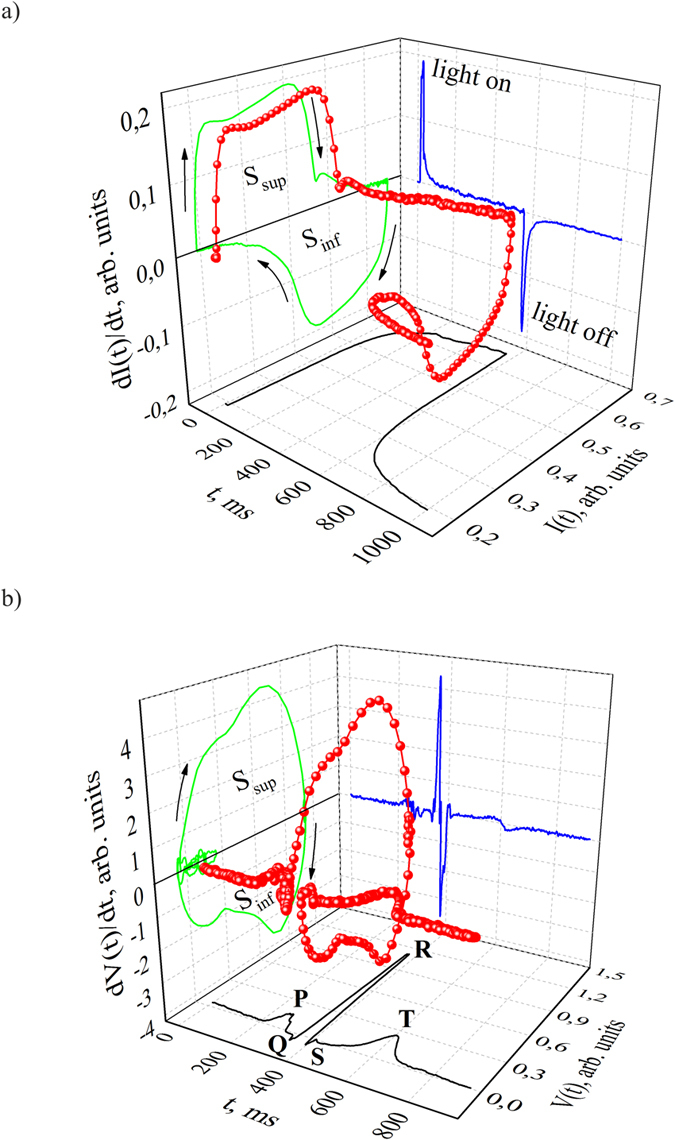
The dynamical state sequences (measured at uniformly time Δ*t* spaced points) in generalized phase space for both *I*(*t*) transient photoresponse from CZT crystal (**a**) and *V*(*t*) ECG signal (**b**).

Main ideas of the approach are considered by example of simple *I*(*t*) transient photoresponse (TPR) and complex *V*(*t*) cardiovascular signal geometrization in phase (time-state-velocity) space. Such signal geometrization is accompanied by *I*(*t*) and *V*(*t*) signals transformation into a sequence of dynamic states (phase space trajectory). This allows natural allocating of segments which are differing in linear density of states (Fig. 1). Interrelations between the dynamic states in these segments are most evident in projections of these trajectories on the phase plane (state-velocity). These projections *I*(*t*) − *dI*(*t*)/*dt* and *V*(*t*) − *dV*(*t*)/*dt* (Fig. 1) are individual phase portraits of bio- and semiconductor sensor and should be considered as a sort of signatures. As you can see, geometrization of different signals is accompanied by the natural decomposition on geometrically ordered segments of constant slope or curvature. Differential-geometric parameters of these segments are individual for each sensor. For an arbitrary signal *x*(*t*) they reflect the dynamic components. Partial contributions *P* are proportional to segment length and slope product or segment length and curvature product^17^. Values of partial contributions are sensitive to external and internal stimuli and fields. Sequence of partial contributions *P*_1_, *P*_2_, *P*_3_, … is the “marker identifier” of operation cycle. Dynamic balance is peculiar to TPR signatures with symmetric configurations. Area *S* enclosed by the *I*(*t*) − *dI*/*dt* TPR signature is very sensitive to the intensity and wavelength of radiation. Area of TPR signature can be statistically represented as a power of possible photoinduced quantum microstates subset 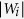
18. From the microscopic point of view, the number of possible quantum microstates *W* can be presented as the statistical weight^19^. Consequently, the power of a microstates subset statistically characterizes the *H* entropy of TPR that is proportional to the natural logarithm of *W*, i.e . 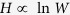. This allows us to analyze the ordering of transient photoresponse from sensor. Therefore, the configuration and area of signature is naturally connected. This allows examining the functioning by the complementary dynamic and statistical methods. This establishes a natural connection between the macroscopic and microscopic characterization of the system. Thus, an integral index of dynamic balance *B*_dyn_ is the area ratio of *S*_sup_ superior and *S*_inf_ inferior parts of signature, i.e. *B*_dyn_ = *S*_sup_/*S*_inf_. TPR entropy increasing during sensor exploitation in extreme conditions is accompanied by violations of the *B*_dyn_ dynamic balance and rearrangement of partial contributions of TPR components.

Phase portraits of human ECG are informative but their analysis is ambiguous^8^. Within the framework of approach the configuration of *V*(*t*) − *dV*(*t*)/*dt* signature for each wave of cardiac cycle can be analyzed dynamically and statistically. Thus, the *V*_*QRS*_(*t*) − *dV*_*QRS*_(*t*)/*dt* signature of QRS-complex includes both signatures of *P*- and *T*-wave of cardiac cycle, i.e. *V*_*P*_(*t*) − *dV*_*P*_(*t*)/*dt* and *V*_T_(*t*) − d*V*_T_(*t*)/*dt* (Fig. 1b). Therefore *W*_*QRS*_ is a dynamic subset includes both *P*- and *T-*subsets of induced microstates. Operations between these subsets and their relationships provide new information which is usually hidden for the other approaches. However, for the system analysis of this information it is necessary to know the cycle of the myocardium, which is genetically inherited.

## Sensor Response Transformation into a Dynamic Event Trajectory

**Figure 2:**
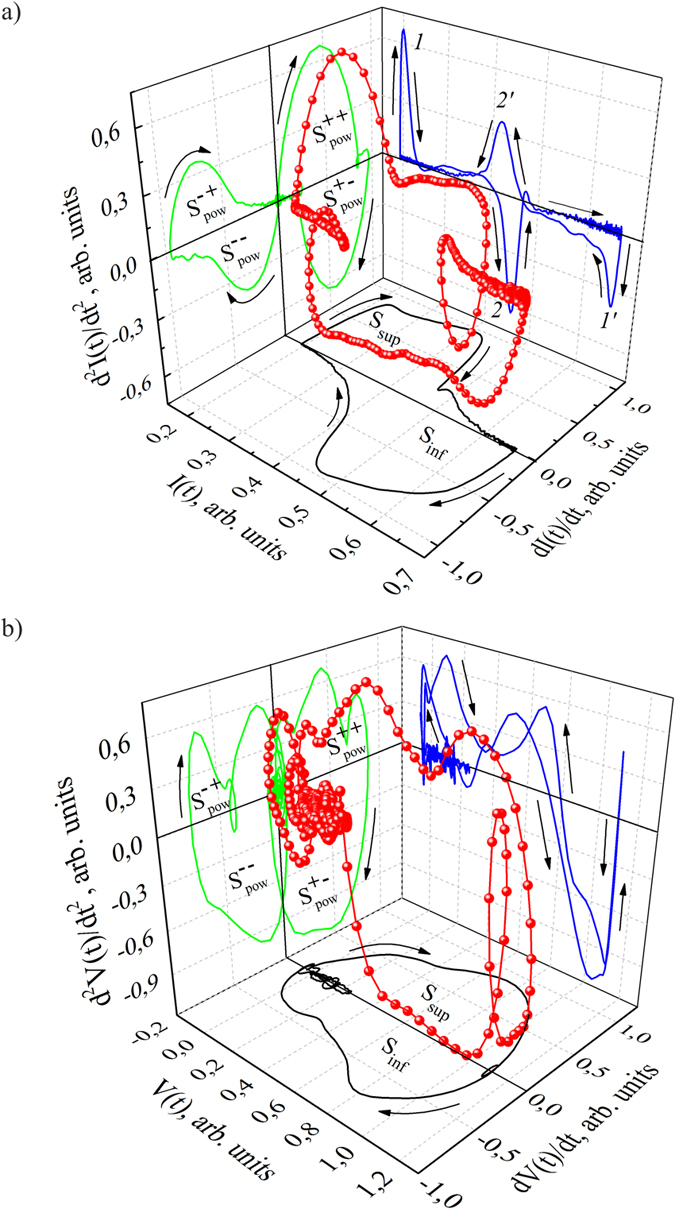
Sensor photoresponse (**a**) and ECG (**b**) as the trajectories of dynamic events in (state - velocity - acceleration) space and projections of these trajectories onto three planes: (state - velocity), (state - acceleration) and (velocity - acceleration). Dots represent dynamic events.

TPR *I*(*t*) geometrization in (state-velocity-acceleration) space allows us to realize a natural transition from dynamic state sequence to dynamic event trajectory. It is possible to increase the number of tools for detection of induced individuality of photoresponse and its analysis^18^. Trajectory consists of curved sections which vary in linear density of dynamic events (Fig. 2). Spatio-temporal correlation between dynamic events is the most evident in the orthogonal projections of trajectory on three planes (state - velocity), (state - acceleration) and (velocity - acceleration). Trajectory projection onto (state - velocity) plane is the *I*(*t*) − *dI*(*t*)/*dt* 1^st^ order signature of TPR which is presented in Section 1. Trajectory projection onto (state - acceleration) plane is the *I*(*t*) − *d*^2^*I*(*t*)/*dt*^2^ 2^nd^ order signature of TPR.

Naturally, extremes on the *I*(*t*) − *d*^2^*I*(*t*)/*dt*^2^ signature coincide with the extremes on *dI*(*t*)/*dt* − *d*^2^*I*(*t*)/*dt*^2^ signatures. The *I*(*t*) − *d*^2^*I*(*t*)/*dt*^2^ signature reflects the energy components of sensor photoresponse (see Fig. 2a). Individuality of antiphase components 1, 2 and 1’, 2’ is shows up in the area ratio between the corresponding extremes, which is energy balance index for them.

Spatio-temporal relationship between the dynamic events determines the configuration of *dI*/*dt* − *d*^2^*I*/*dt*^2^ 2^nd^ order signature. Signature configuration is located at 4 quadrants of (velocity - acceleration) plane and represents relationship between *dI*/*dt* and *d*^2^*I*/*dt*^2^ dynamical variables in main phases of TRP (Fig. 2a, “+ +”, “+ −”, “− −” and “− +” quadrants). Technology inherited defect structure of a crystal determines the complex frequency spectra of resonant vibrations^20^,^21^. It also affects the configuration of the *dI*/*dt* − *d*^2^*I*/*dt*^2^ signature. It should be noted that parameters of coupled resonant vibrations are sensitive to photoexcitation.

The areas covered by the *dI*/*dt* − *d*^2^*I*/*dt*^2^ signature in each quadrant represent the power of basic phases of the bicycle^22^. Indeed, transition to a new dynamic variable *Y* = *dI*/*dt* allows us to convert the *dI*/*dt* − *d*^2^*I*/*dt*^2^ 2^nd^ order TPR signature into the *Y*(*t*) − *dY*/*dt* 1^st^ order signature. Configuration of the *Y*(*t*) − *dY*/*dt* signature occupies 4 quadrants and represents the basic phases of functioning cycle. Therefore, the *dI*/*dt* − *d*^2^*I*/*dt*^2^ TPR signature is a natural geometrical model of the cycle control. The dimensionless indexes *B*_ij_ of the power balance between the basic phases of TPR characterize the spatial-temporal coherence of dynamical processes. The *B*_ij_ indexes are the ratios between areas covered by the *dI*/*dt* − *d*^*2*^*I*/*dt*^2^ signature in each quadrant, e.g. 
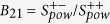
, etc. In a square matrix of the *B*_ij_ indexes represents relations between the basic phases of sensor functioning (Table 1). Individuality of sensor functioning is most evident in matrix (Table 1).

The *dV*/*dt* − *d*^2^*V*/*dt*^2^ signature configuration is a geometrical model of CVS functioning (Fig. 2b). It revealed the variety of geometric patterns in myocardium cycle. It is established the high sensitivity of *B*_ij_ indexes of cardicycle to external and internal factors (magnetic storms, drugs, food, etc.). All of this opens up new opportunities for the system analysis of cardiac cycle in contrast to known methods. For instance, atlas creation for the typical geometrical models of CVS functioning simplifies not only their parametric identification and classification but also express diagnostics.

## Integral Individuality of System Functioning

**Figure 3:**
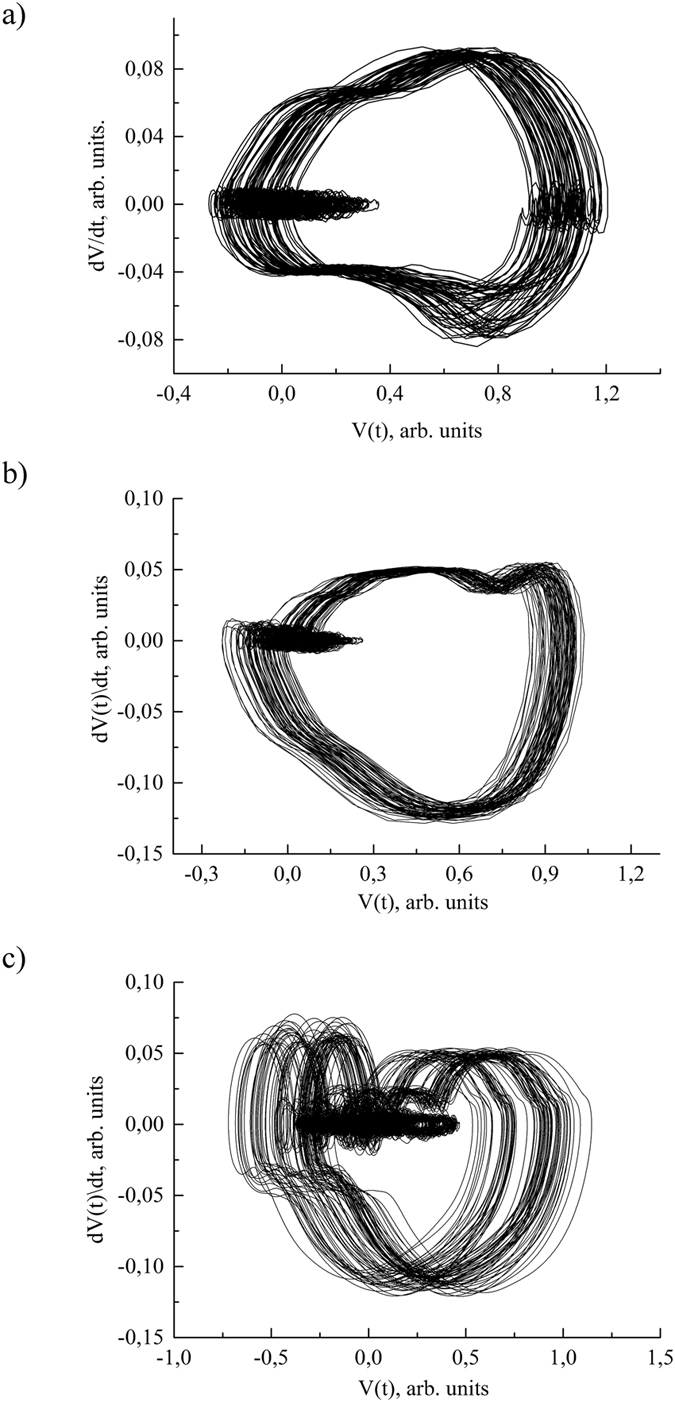
The 1^st^ order signature packages for ECG from three monitored people.

**Figure 4:**
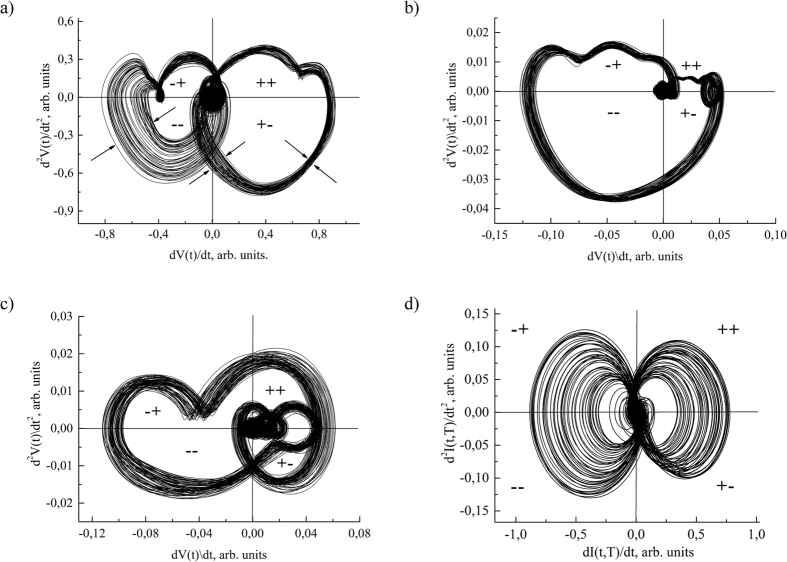
The 2^nd^ order signature packages for ECG (**a–c**) from three monitored people and TPR from semiconductor sensor (**d**). Corresponding 1^st^ order signatures of QRS-complexes are shown in Fig. 3.

Identification of inherited functioning individuality is promoted by the fruitful idea of the ECG^23^ (Fig. 3) and TPR^11^ sequences presentation in a package form. The series of highly stable TPR cycles from sensor are shown in signature package as an attractor^23^,^24^. The characteristic features of the stable sensor functioning are: reversibility of cycles and their dynamic balance. These characteristic features are interrelated and combined with the minimum of covered area, i.e. entropy minimum of TPR. They correspond to thermodynamic criteria of cycle reversibility Δ*H* → 0, where *H*-entropy. Exploitation of these sensors in extreme conditions is accompanied by dynamical and energy imbalance of antiphase processes and can also lead to local distortion of the *I*(*t*) − *dI*/*dt* (Fig. 3a–c) and *dI*/*dt* − *d*^2^*I*/*dt*^2^ (Fig. 4a–c) TPR signatures within the packages. However electroacoustic treatment at frequencies corresponding to a certain piezoelectric resonant vibrations improves the sensor’s functional characteristics. Temperature treatment should be performed at *T*_*i*_ temperature, where *T*_*i*_ is a certain temperature at which TPR signature configuration becomes most symmetrical (Fig. 4d)^25^.

Package presentation of physiological signal is a quite effective to analyze integral individuality of system functioning. Analysis of more than 50 packages of the *V*(*t*) − *dV*/*dt* signature of QRS-complex shows diversity of nature of dynamic restructuring. The *V*(*t*) − *dV*/*dt* signature packages for three monitored people that have features of myocardium functionality altering^26^ are showed in Fig. 3. The 1^st^ order signature restructuring over the package is qualitatively presented in:

1. Phase trajectories distribution character within the signature package (homogeneous, heterogeneous, step-like *et al.*).
2. Appearance of local variations Δ*V*(*t*) in signature over the cycle under influence of stress factor that is proportional to Kolmogorov entropy^27^.

Functional individuality is quantitatively manifested in entropy and entropy production time dependences, i.e. *H*(*t*) and *dH*(*t*)/*dt* The *H*(*t*) and *dH*(*t*)/*dt* time dependences converting into package of integro-differential *H*-signatures *H*(*t*) − *dH*(*t*)/*dt* leads to a typical chaos-gram^28^. However, in contrast to chaos-gram the package of *H*-signatures allow us to study individuality of human CVS. A duality of biological order which represents the relation between the structures and functioning processes^29^ is most manifested in *H*-signature packages. It can be assumed that *H*-signature packages with a high enough resolution will provide us information about hidden relations between human body subsystems. Partly they appear in nature rearrangement of the *dV*/*dt* − *d*^2^*V*/*dt*^2^ signatures of QRS-complex in the package (Fig. 4a–c).

CVS control system rearrangement can be analyzed dynamically (signature configuration change) and statistically (density of trajectories and area covered by each quadrant). It can be assumed that information about individual structure of interrelations is hidden in the character of evolution of *dV*/*dt* − *d*^2^*V*/*dt*^2^ signature configuration (Fig. 4a–c) and area covered by *dV*/*dt* − *d*^2^*V*/*dt*^2^ signature of Q, R and S waves. Individuality of adaptation processes is the most evident in evolution of *dV*/*dt* − *d*^2^*V*/*dt*^2^ signature configuration.

Package of the *dV*/*dt* − *d*^2^*V*/*dt*^2^ signatures can be considered as subsets of operation cycles. Integrated individuality manifests in restructuring of operation cycles. Obviously, the systemically important function (inherited scenario of system functioning) is hidden in operation cycles and relationship between them. This is indicated by results of comparative analysis of ECG signature of 7 relatives. It turned out that configuration of the 1^st^ and 2^nd^ order signature for 1, 2 and 3 leads are similar only for father (70 years old), daughter (41) and grandson (8 years). These signatures are characterized by similar integrative indexes (*B*_ij_, *H* etc.) Package of these signatures also identified the same characteristic features of signature restructuring.

It was found that package of 1^st^ and 2^nd^ order signatures ECG contains information on the main indexes of CVS (level of functioning, functional reserve and degree of tension of regulatory mechanisms). Today, scientists use different methods of diagnosis to determine the main indexes of CVS. The versatility of parameters, indexes and criteria allows avoiding the ambiguity during analysis of complex study results in ergonomics, medicine, sports, etc. Natural geometrization of signals from different type of sensors simplifies analysis of both consistency and compatibility in manned subsystems. Natural cycles of biosystem operation for cybernetic systems are of particular interest for cyber physical system developers.

## Conclusions

At different scale levels of signals the dynamic, energetic and cybernetic aspects of biological and physical systems functioning are hidden. They manifested after converting any cyclic signal *X*(*t*) and its derivatives in the trajectory of dynamic events. In fact, the trajectory is a geometric interpretation of variational Hamilton’s principle of least action. As a result of such geometrization the natural decomposition of signal onto geometrically ordered sections that reflect its dynamic and energetic components is carried out. Universal differential-geometrical parameters of these sections (length, slope and curvature) are mapped to the physical values (state, velocity and acceleration). Therefore, the orthogonal projection of the trajectory are *X*(*t*) − *dX*/*dt*, *X*(*t*) − *d*^2^*X*/*dt*^2^ and *dX*/*dt* − *d*^2^*X*/*dt*^2^ signatures. Individual configuration of the signatures is naturally combined with a statistical regularity. Indeed, the spatial and temporal correlation of dynamic events are converted into geometrically ordered sections (dynamic and energy components of *X*(*t*) − *dX*/*dt*, *X*(*t*) − *d*^2^*X*/*dt*^2^ signature configurations). The nature of relationship between *dX*/*dt* and *d*^2^*X*/*dt*^2^ components determines individual cycle of system functioning that displayed by the *dX*/*dt* − *d*^2^*X*/*dt*^2^ signature. The matrix of power balance indexes is proposed for analysis of the basic phases of functioning cycle.

Individuality is most evident in the character of rearrangement of system functioning cycle and manifested in a packet presentation of cyclic signal signatures. Both configuration and area changes of signatures are interrelated which allows to analyze functioning artifacts. Thus, various stress factors rise to local instability Δ*X* and imbalances of the main phases of functioning cycle. Presentation of area covered by the *X*(*t*) − *dX*/*dt* signature as a subset of microstates allows to provide entropy analysis of system functioning. Therefore, the character of signature configuration and area of changes in a natural way manifested the relation between dynamic and statistical regularities in system functioning. The results of integration of signatures in the package (*H*(*t*) dependence) and its derivative *dH*/*dt* are informative. Consistent signature configuration change and entropy H is peculiar to biological systems. For their studies the integro-differential *H* signature *H*(*t*) − *dH*/*dt* and their packages are proposed for the first time. They naturally represent biological order based on the inherited relation of structures and functions. In essence, the proposed approach is a kind of bridge between the macroscopic and microscopic description of systems functioning.

The approach has also proved effective for the signature analysis of other functional characteristics of sensors. Their signatures displayed the influence of “frozen” dynamics of defect structure onto individuality of functional characteristics of semiconductor sensors. For example, spectral signatures^30^ and temperature^31^,^32^ PR dependencies allowed to establish a complex energy spectrum of defects which had previously been identified by various methods. The proposed approach is also compatible with other signal processing methods. Thus, the transformation of wavelet spectrograms of photoresponse to wavelet signatures at different scales allowed to reveal usually hidden information by means of universal tools of approach^33^. Search studies have shown that higher-order signatures are promising for the analysis of multi-scale signals.

In general, our approach and universal tools for its implementation provide new opportunities for diagnostic systems, harmonization of human-computer interaction (human-computer interaction) and ensure intercomputer interaction (machine-machine interaction) in cyberphysical systems. Obviously, application of approach tools will simplify the control of consistency in subsystems of technical systems.

## Additional Information

**How to cite this article**: Mygal, V. P. *et al.* An interdisciplinary approach to study individuality in biological and physical systems functioning. *Sci. Rep.* **6**, 29512; doi: 10.1038/srep29512 (2016).

## Figures and Tables

**Table 1:** Square matrix of the power balance indexes between the basic phases of the functioning bicycle.

Quadrants	“+ +”	“+ −”	“− −”	“− +”
“+ +”	1	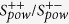	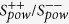	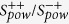
“+ −”	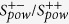	1	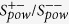	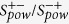
“− −”	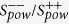	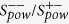	1	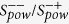
“− +”	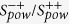	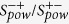	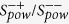	_1_
